# Naturally Occurring Mutations in HIV-1 CRF01_AE Capsid Affect Viral Sensitivity to Restriction Factors

**DOI:** 10.1089/aid.2017.0212

**Published:** 2018-04-01

**Authors:** Emi E. Nakayama, Akatsuki Saito, Tahmina Sultana, Zhuan Jin, Kyotaro Nohata, Masato Shibata, Miho Hosoi, Kazushi Motomura, Tatsuo Shioda, Somchai Sangkitporn, Ruangchai Loket, Siriphan Saeng-aroon

**Affiliations:** ^1^ Department of Viral Infections, Research Institute for Microbial Diseases, Osaka University, Osaka, Japan.; ^2^ Thailand-Japan Research Collaboration Center on Emerging and Re-Emerging Infections (RCC-ERI), Osaka University, Nonthaburi, Thailand.; ^3^ National Institute of Health, Department of Medical Science, Ministry of Public Health, Nonthaburi, Thailand.

**Keywords:** TRIM5α, MxB, CRF01_AE, capsid

## Abstract

TRIM5α and MxB are known as restriction factors that inhibit the early step of intracellular HIV-1 replication cycle. Both factors are believed to interact with the incoming virus core to suppress HIV-1 infection. The extreme diversity of HIV-1 is thought to be a consequence of its propensity to mutate to escape immune responses and host restriction factors. We recently determined the capsid sequences for 144 HIV-1 CRF01_AE viruses obtained in Thailand from 2005 to 2011. In this study, we further analyzed the amino acid variations among the capsid sequences of 204 HIV-1 CRF01_AE obtained in Thailand and China, including 84 of the aforementioned 144 viruses, to detect mutations permitting escape from restriction by host factors. We found a characteristic combination of E79D, V83T, and H87Q in sequences from Chinese viruses and subsequently showed that this combination conferred partial resistance to MxB. Interestingly, this combination conferred resistance to human TRIM5α as well. The H87Q mutation alone conferred resistance to MxB in the CRF01_AE background, but not in subtype B virus. In contrast, the H87Q mutation alone conferred resistance to human TRIM5α in both the CFR01_AE and subtype B backgrounds. BLAST analysis revealed the presence of the E79D, V83T, and H87Q combination in CRF01_AE viruses isolated not only in China but also in many other countries. Although the mechanistic details as well as precise role of MxB antiviral activity in infected individuals remain to be clarified, our data suggest an interaction between MxB and the HIV-1 capsid *in vivo*.

## Introduction

Four major restriction factors capable of suppressing HIV replication have been reported: human ApoB mRNA editing catalytic subunit (APOBEC) 3G^1–3^; human Tetherin, also known as BST2 or CD317^4,5^; human SAMHD1 (a cellular protein SAM and HD domain-containing protein)^6–8^; and monkey TRIM5α/TRIMCyp.^9,10^ Recently, human MxB (but not MxA) was reported as another restriction factor induced by type 1 interferon (IFN). Monkey TRIM5αs exhibit relatively strong anti-HIV-1 effects, while the anti-HIV-1 activity of human TRIM5αs is comparatively weaker.^11^ Human MxB^12^ exhibits a strong anti-HIV-1 effect, while MxA does not. The precise mechanism of MxB-mediated inhibition of HIV-1 is unknown,^13,14^ but it is clear that the expression of MxB is induced by type 1 IFN.^12,15^ Among these restriction factors, APOBEC3G, BST2, and SAMHD1 are degraded through proteasomal pathways, and their antiviral activities are inhibited in the presence of viral proteins HIV-1 vif,^16–18^ HIV-1 vpu,^4,5^ and HIV-2 vpx,^8,19^ respectively. In contrast, HIV accessory proteins are unable to counteract MxB and TRIM5α, since HIV interacts with those factors through the viral capsid.^20–22^ However, it is currently unknown whether HIV-1 is able to evolve to evade MxB-mediated inhibition. One way to address this question is to determine whether MxB-resistant HIV-1 strains exist in infected individuals. Therefore, in this study, we analyzed 204 HIV-1 sequences, most of which were derived from recent cases infected through sexual contact. We show, in this study, that characteristic mutations found in isolates obtained in China indeed confer partial resistance to MxB.

## Materials and Methods

### Cell culture

293T, a human kidney adherent cell line, was cultured in Dulbecco's modified Eagle's medium supplemented with 10% heat-inactivated fetal bovine serum (FBS) at 37°C with 5% CO_2_. MT4, a human CD4^+^ T cell line immortalized by human T cell leukemia virus type 1,^23^ was maintained in RPMI 1640 medium containing 10% FBS at 37°C with 5% CO_2_.

### Reporter-expressing viruses

For the introduction of point mutations in the capsid (CA)-encoding region, we used site-directed mutagenesis employing mutagenized overlapping primers. *Bss*HII-*Apa*I fragments that encode matrix (MA) and CA were transferred into NL4-3-Luc-R-E (NIH AIDS Research and Reference Reagent Program) or pMSMnG^24^ to generate the corresponding luciferase reporter and green fluorescence protein (GFP) vectors, respectively. To recover the vesicular stomatitis virus glycoprotein (VSV-G)-pseudotyped luciferase-expressing viruses, 293T cells were cotransfected with 12 μg of reporter plasmid and 5 μg of VSV-G-expressing plasmid (pMD2G).^25^ The GFP-expressing N-MLV and B-MLV were obtained by transfection of the plasmid.^26^

### Recombinant Sendai viruses

Sendai viruses (SeV) encoding human TRIM5α or cynomolgus monkey (CM) TRIM5α without the SPRY domain [CM-SPRY (−)] were described previously.^21,27,28^ Sequences encoding human MxB were purchased from Origene and used as a template to generate a recombinant SeV vector expressing MxB with an HA-tag in its C-terminus.

### Single-round infection assay

MT4 cells were infected with SeV encoding human MxB, human TRIM5α, or CM-SPRY (−) as previously described, and the cells were then superinfected with VSV-G-pseudotyped HIV-1 clones harboring a luciferase reporter gene. Three days after infection, the luciferase activities in infected cells were measured using the Bright-Glo™ Luciferase Assay (Promega, Madison, WI) according to the manufacturer's instructions. The infectivity of a clone was measured by dividing the luciferase activity of that clone in the presence of CM-SPRY (−) by the luciferase activity of wild-type NL4-3-Luc-R-E- in the presence of CM-SPRY (−). To determine the resistance of a given clone to human MxB or TRIM5α, the luciferase activity of that clone in the presence of human MxB or TRIM5α, respectively, was divided by the luciferase activity of that clone in the presence of CM-SPRY (−).

### Interferon treatment

We treated MT4 cells and THP-1 cells for 16 h with 200 U/ml of interferon (IFN)-β and then superinfected with VSV-G-psudotyped GFP-expressing HIV-1. For MLV infection, we infected canine Cf2Th cells, which did not express endogenous TRIM5 protein, with SeV expressing TRIM5α or CM-SPRY (−). Nine hours after infection, cells were superinfected with N-MLV expressing GFP, B-MLV expressing GFP, or HIV-1 expressing GFP. The GFP-positive cells were counted by EC800 cell analyzer (SONY).

### Western blot analysis of cell lysates

Pelleted cells were resuspended in 1 × NuPAGE LDS sample buffer (Thermo) containing 2% β-mercaptoethanol. Expression of endogenous MxB in IFN-β-treated cells was evaluated with Western blot using goat anti-MxB polyclonal antibody (SantaCruz Biotech) followed by HRP-conjugated donkey anti-goat IgG antibody (SantaCruz Biotech). Expression of HA-tagged host factors in SeV-infected MT4 cells was confirmed by Western blot using rat anti-HA monoclonal antibody (Roche Diagnostics) followed by HRP-conjugated goat anti-rat IgG antibody (American Qualex). The membranes were probed with rabbit anti-β-actin polyclonal antibody (Thermo) as a loading control. Chemiluminescence was detected using Chemi-Lumi One Ultra reagent (Nacalai Tesque) according to manufacturer's instructions.

### Western blot analysis of virions

293T cells were transfected with pNL4-3.Luc.R-E- plasmids using TransIT-LT1 Transfection Reagent (Clontech) according to the manufacturer's instructions. It should be noted that cells were transfected in the absence of the VSV-G protein to prevent infection of cells with newly produced virions. Equal volume of culture supernatants (900 μl) was layered onto 500 μl of 20% sucrose in phosphate-buffered saline and centrifuged at 20,000 × *g* for 2 hours at 4°C. Pelleted virions were resuspended in 1 × NuPAGE LDS sample buffer containing 2% β-mercaptoethanol. Lysed virions were subjected to Western blot. The p24 CA protein was probed with mouse anti-p24 monoclonal antibody (Abcam) followed by HRP-conjugated goat anti-mouse IgG antibody (KPL). To check transfection efficiency, 293T cells were lysed with cell culture lysis reagent (Promega) and used to measure luciferase activity with a luciferase assay kit (Promega) on a luminometer.

## Results

### Sequences of recently infected HIV-1 CRF01_AE

We previously explored the molecular epidemiology of HIV in female sex workers in Thailand recently infected with HIV-1.^29^ That analysis used samples collected by the National HIV Drug Resistance Surveillance Program in 2005, 2007, 2009, and 2011. The genetic subtype of each isolate was determined in all 159 specimens (NCBI accession No. LC114665-LC114820). Neighbor-joining analysis placed 90.6% (144/159) of the sequences in HIV-1 CRF01_AE. Among the HIV-1 CRF01_AE viruses, we identified 83 sequences that lacked ambiguities or polymorphisms and converted these sequences to amino acid sequences. The predicted protein sequences corresponded to amino acid positions 23 to 210 of the capsid. To provide sequences from the earlier outbreak in Thailand, we collected (from the NCBI database) capsid protein sequences from 37 and 5 CRF01_AE and CRF01/B clones, respectively, which had been isolated in Thailand before 2000 (Supplementary Table S1; Supplementary Data are available online at www.liebertpub.com/aid).

**FIG. 1.**
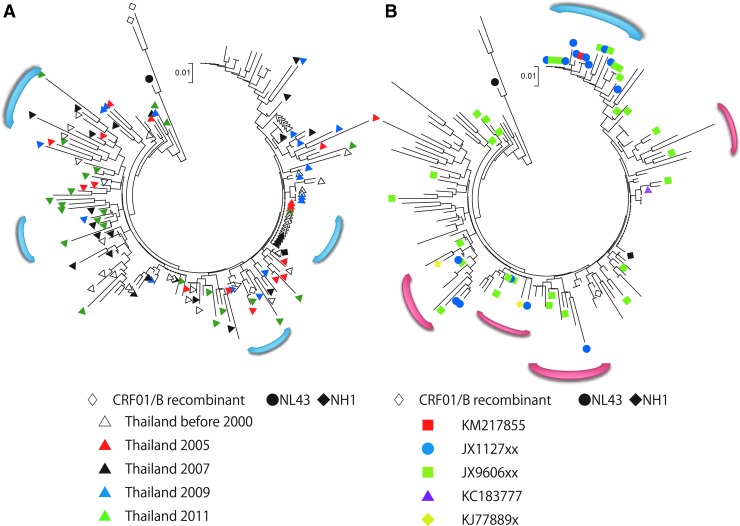
Phylogenetic tree of HIV-1 capsid amino acid sequences. Thai **(A)** and Chinese **(B)** sequences are highlighted in a phylogenetic tree of all the analyzed sequences. The *black circle* and *black diamond* indicate NL4-3 (subtype B) and NH1 (CRF01_AE), respectively. *Blue parentheses* indicate clusters consisting of Thai **(A)** or Chinese **(B)** sequences only. *Red parentheses* indicate clusters consisting of both Thai and Chinese sequences. For **(A)**, *unfilled triangles* are sequences reported before 2000; *red*, *black*, *blue*, and *green triangles* are sequences collected in 2005, 2007, 2009, and 2011, respectively. For **(B)**, sequences are designated by their NCBI accession numbers, with x's indicating digits that varied depending on specific isolate number (see Supplementary Table S2). *Unfilled diamonds* indicate the CRF01/B recombinants.

In addition, we collected (from the NCBI database) capsid protein sequences from 49 CRF01_AE clones obtained from a recent outbreak in China among men having sex with men (MSM) (Supplementary Table S2). We also incorporated capsid protein sequences from the standard HIV strains NL4-3 (subtype B, NCBI accession No. AF324493) and JP93-NH1 (NH1, CRF01_AE, NCBI accession No. AB052995). In total, 176 capsid protein sequences were used for phylogenetic tree construction by the neighbor-joining method. To ensure that the analyzed sequences were of consistent length, we used sequences encompassing capsid protein residue 23 to 210 for alignment. Based on the phylogenetic tree analysis (Fig. 1A, B), these HIV isolates constituted several Thai clusters and one Chinese cluster. Several clusters were composed of both Thai and Chinese sequences. These results indicated that there have been several independent transmissions of HIV-1 CRF01_AE from Thailand to China. Among the Thai sequences, strains isolated in 1999 and 2005 to 2011 belonged to a shared cluster.

**FIG. 2.**
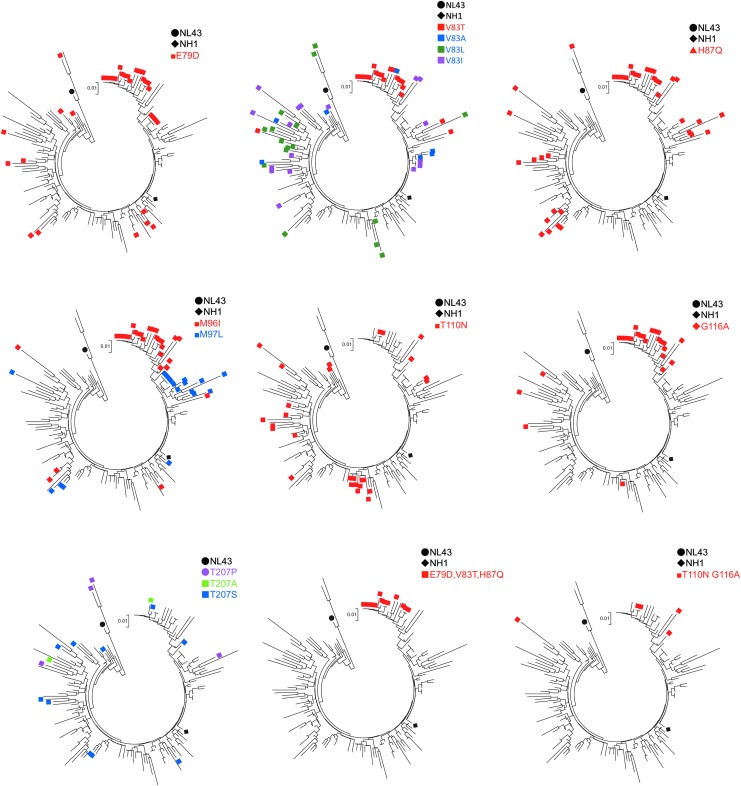
HIV-1 capsid amino acid variations in CRF01_AE. In each tree, sequences possessing minor amino acid residues at the specified positions are labeled. The *black circle* and *diamond* indicate NL4-3 (subtype B) and NH1 (CRF01_AE), respectively.

**FIG. 3.**
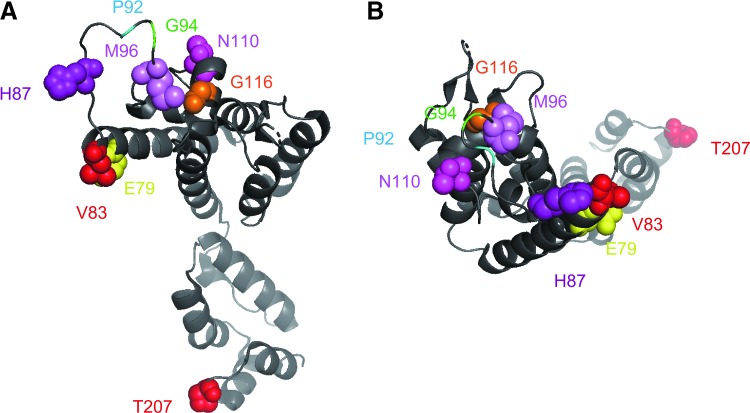
HIV-1 capsid monomer 3D structure model. Side **(A)** and top **(B)** views of the capsid monomer based on pdb file 4XFY were visualized by Mac Pymol version 2.0. Amino acid positions 79, 83, 87, 96, 110, 116, and 207 are indicated by spheres. The G94S substitution in 93JP-NH1 and major difference between subtype B and CRF01_AE at position 92 are also shown.

When we looked at the each amino acid position, we observed that positions 79, 83, 87, 96, 110, 116, 120, 128, 149, 178, and 207 are highly polymorphic. Notably, the E79D, V83T, and H87Q mutations tended to be inherited together, and the 79D, 83T, and 87Q combination (DTQ) were found exclusively in the Chinese sequences analyzed in this study (Fig. 2). In addition, the M96I, T110N, and G116A mutations exhibited partial linkage with DTQ. In contrast, the T207S and T207P mutations were not inherited with DTQ or other mutations (Fig. 2). The positions of amino acid that we described in this article were shown on the 3D structure on the pdb file 4XFY (Fig. 3).

### The effect of capsid mutations on restriction by human MxB and TRIM5α

**FIG. 4.**
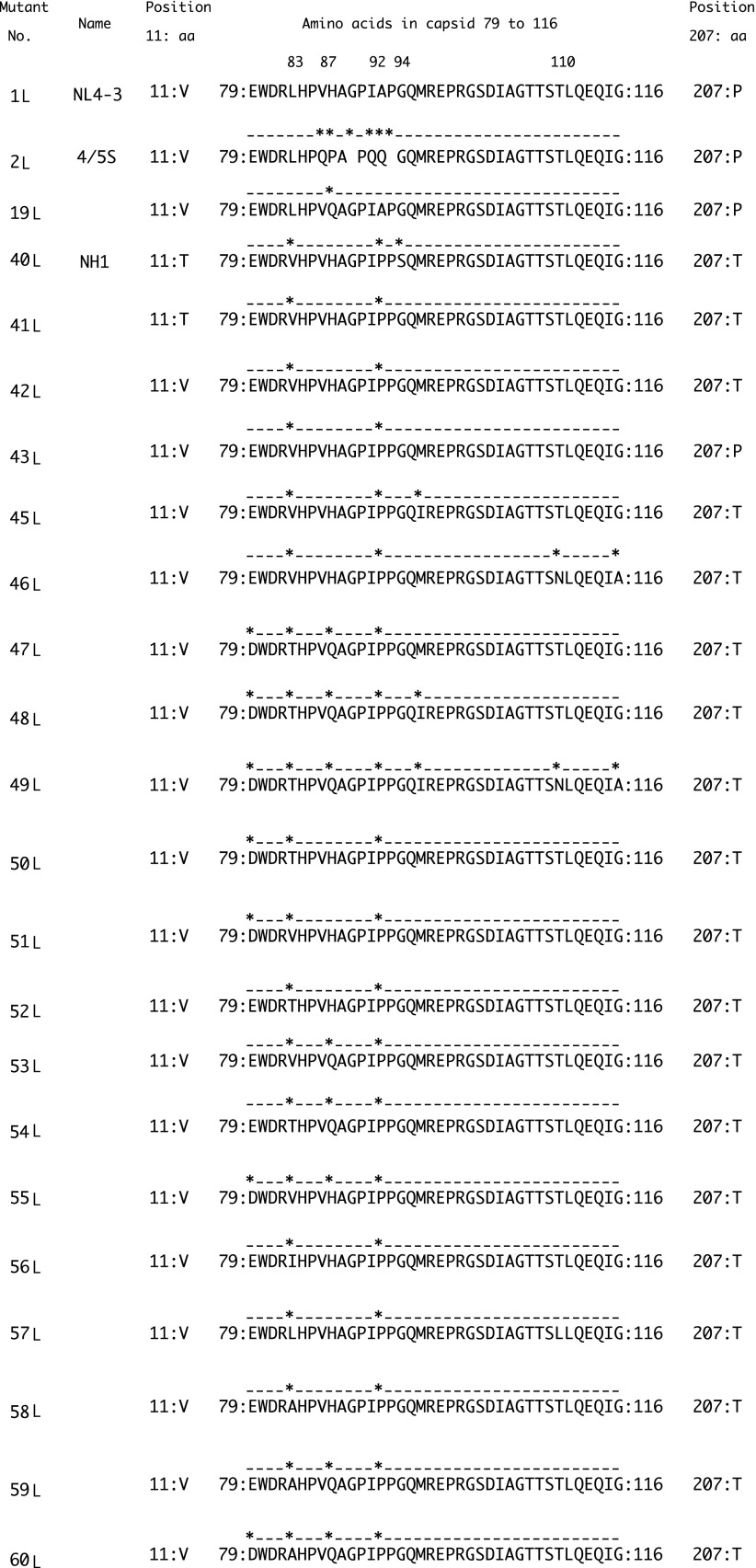
Alignment of a segment of the capsid protein of HIV-1 constructs generated in this study. *Dots* and *asterisks* denote amino acid residues identical to and different from, respectively, those of NL4-3. The numbers indicate the amino acid position in the NL4-3 capsid.

To examine the aforementioned characteristic mutations found in CRF01_AE, we constructed a chimeric molecular clone based on NL-Luc-R-E-. First, we replaced the region encoding capsid and p6 (nucleotide position 1182 to 1970 in NL4-3; Fig. 4) with the corresponding region of CRF01_AE molecular clone 93JP-NH1^30^; the resulting clone was designated 40L.

**FIG. 5.**
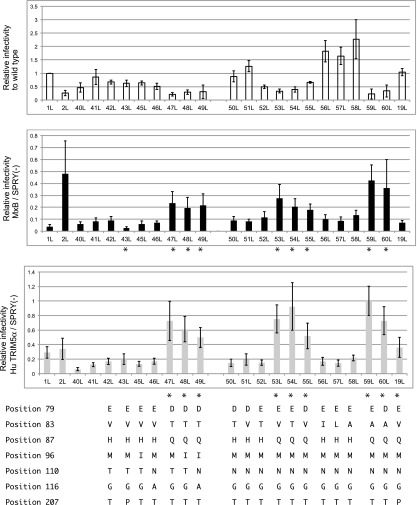
Viral infectivity in the absence or presence of human MxB and TRIM5α in a single-round infection assay. MT4 cells expressing human MxB, TRIM5α, or CM-SPRY (−) were superinfected with vesicular stomatitis virus glycoprotein-pseudotyped HIV-1 derivatives with a luciferase reporter gene. The infectivity of each clone was defined by normalizing the luciferase activity of the clone to the luciferase activity of the wild-type NL4-3-Luc-R-E- in the presence of non-functional TRIM5α, CM-SPRY (−) (*white bars*). To quantify the sensitivity of each clone to human MxB (*black bars*) or TRIM5α (*gray bars*), luciferase activity of the clone in the presence of human MxB or TRIM5α, respectively, was normalized to the luciferase activity of the respective clone in the presence of CM-SPRY (−). Results are shown as the mean ± SD of at least three independent experiments. *Asterisks* indicate a statistically significant (*p* < .05) difference as assessed by a two-tailed unpaired Student's *t*-test comparison to clone 42L. Amino acid residues at positions 79, 83, 87, 96, 110, 116, and 207 of the indicated clones are shown at the *bottom*. CM, cynomolgus monkey; SD, standard deviation.

We infected a CD4+ T cell line, MT4, with recombinant SeV expressing MxB or human TRIM5α.^27^ SeV expressing CM TRIM5 lacking the PRYSPRY domain, CM SPRY (−), served as a control for SeV infection in the absence of restriction factors.^21^ Unfortunately, the infectivity of the 40L virus encoding the CRF01_AE capsid was relatively low, even in the MT4 cells infected with SeV expressing CM SPRY (−) (Fig. 5, Supplementary Figs. S1 and S2). To address this challenge, we added an S94G mutation to 40L, yielding a clone that was designated as 41L; we incorporated this allele because we noted that the 93JP-NH1 clone alone possessed a rare mutation encoding S at amino acid 94 of the capsid among 176 capsid sequences we analyzed. As expected, inclusion of the S94G substitution in 41L restored infectivity compared to 40L (Fig. 5).

We next introduced a T11V mutation to 41L, yielding a clone that was designated as 42L; we incorporated this allele because we noted that the 93JP-NH1 clone possessed a minor mutation encoding T at amino acid 11 of the capsid. Four percentage of CRF01_AE sequences analyzed in this study possessed T. The capsid amino acid sequence of 42L (position 23 to 210) was identical to the 13 sequences from Thailand (AY945732, AY945718, AY358071, AY358064, AY358057, AY358052, AY358051, LC114701, LC114708, LC114740, LC14771, LC114670, and LC114672). In contrast to the case with S94G, the T11V mutation did not apparently change the clone's infectivity.

We subsequently introduced individual mutations or combinations of mutations at positions 78, 83, 87, 96, 110, 116, and 207 of 42L, as summarized in Figure 4; the resulting clones were designated 43L through 60L. The amino acid sequence from position 23 to 210 of 49L was identical to the sequences from China (KM217855 and JX112796).

**FIG. 6.**
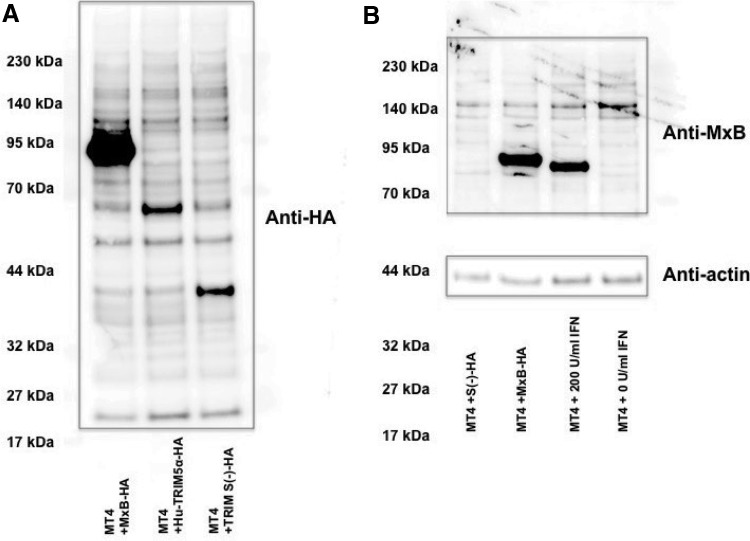
Western blot analysis of restriction factors. **(A)** Lysates of MT4 cells infected with recombinant SeV expressing HA-tagged MxB, human TRIM5α, or CM-SPRY (−) were subjected to sodium dodecyl sulfate–polyacrylamide gel electrophoresis followed by Western blot analysis. Transferred proteins in membrane were visualized by an antibody against HA. **(B)** Lysate of MT4 cells treated with 200 U/ml of IFN-β for 16 h were lysed and visualized by Western blot with an antibody against MxB. An antibody against actin was used for internal control. MT4 cells infected with SeV expressing MxB or CM-SPRY (−) were lysed 16 h after infection. IFN, interferon; SeV, Sendai viruses.

When we examined infection in cells co-infected with an MxB-expressing SeV, the reporter virus with NL4-3 capsid (1L) was suppressed nearly 30-fold, as we expected. In contrast, a reporter virus in which the loop between α-helices 4 and 5 of the NL4-3 capsid was replaced with the corresponding loop of SIVmac^31^ (2L) showed resistance to MxB-mediated restriction, since 2L showed only twofold change in viral titer in the presence of MxB (Fig. 5). This resistance was seen despite the high level of MxB expression observed in SeV-infected cells (Fig. 6). This result was in good agreement with a previous report indicating that HIV-1 was more sensitive to MxB than was SIVmac.^12^

In a previous work, the P207S mutation seen in subtype B was reported to endow HIV with resistance to human MxB.^32^ In CRF01_AE, the major amino acid at the position 207 is T, but the consequence of 207T in MxB resistance was not known. When we introduced a T207P mutation into 42L, the resulting virus (designated clone 43L) showed an increased sensitivity to MxB, (*p* = .04). The V86A, H87Q, and A92P mutations present in subtype B were previously reported to endow HIV with resistance to human MxB.^33^ In this study, 42L (which possesses the A92P mutation) did not show apparent resistance to human MxB (Fig. 5). Mutation of 42L to include M96I (yielding clone 45L) or T110N and G116A (yielding clone 46L) also did not reveal apparent changes in virus sensitivity to MxB. However, the introduction of the combination of the DTQ mutations (at position 79, 83, and 87) with A92P (clone 47L); A92P and M96I (clone 48L); or A92P, M96I, T110N, and G116A (clone 49L) conferred resistance to MxB, with a significant difference detected between 42L and 47L, between 42L and 48L and between 42L and 49L (*p* < .05 by a two-tailed unpaired Student's *t*-test).

**FIG. 7.**
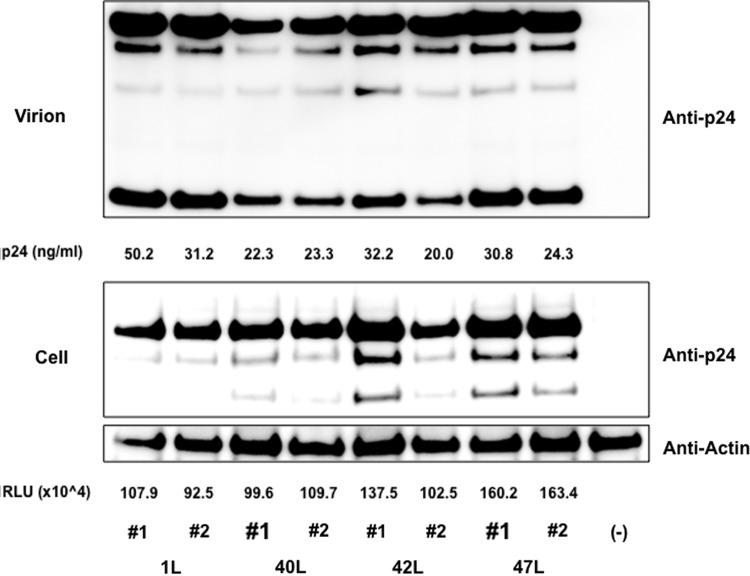
Western blot analysis of capsid in virion and producing cells. 293T cells were transfected with pNL-Luc-R-E- plasmid (1L) or mutants 40L, 42L and 47L. Cell lysate of 293T cells and supernatant was subjected to Western blot and capsid proteins were visualized by anti-CA monoclonal antibody. The amounts of p24 antigen in the supernatant and luciferase counts (RLU) in the transfected cells are shown. The experiments were performed in duplicate.

Notably, the DTQ mutation also yielded resistance to human TRIM5α, although the virus infectivity itself was inferred with DTQ mutation (Fig. 5). In our SeV-expressing system, human TRIM5α was readily detected in Western blot (Fig. 6) and could potently suppress N-MLV, while B-MLV was highly resistant to human TRIM5α (Supplementary Fig. S3). It should be also noted here that Western blot analyses of capsid protein in virion and plasmid-transfected 293 T cells revealed no obvious abnormality in Gag processing of 40L, 42L, and 47L viruses (Fig. 7).

To specify the contribution of each amino acid substitution of DTQ, we introduced the component mutations alone or in pairs into 42L (yielding clones 50L–60L). Resistance to MxB and human TRIM5α was observed when we introduced H87Q alone or in combination with the V83T or E79D mutation (clones 53L, 54L, and 55L, respectively) (*p* < .05 by a two-tailed unpaired Student's *t*-test). The amino acid residues encoded by Thai isolates at position 83 were T, I, L, or A. We therefore introduced mutations to encode each of these amino acid residues at position 83 (clones 52L, 56L, 57L, and 58L, respectively). We did not detect any significant change in the resistance to MxB with these mutations at position 83 alone, but we found that the combination of H87Q with a mutation at position 83 or 79 (clones 59L and 60L, respectively) conferred resistance to MxB.

Previously, the H87Q mutation in subtype B was reported to contribute to the escape of HIV-1 from MxB^33^ and human TRIM5α^34^-mediated restriction. Similar findings for H87 have been published for Clade C HIV-1.^35^ We attempted to extend that previous analysis by comparing the effect of H87Q alone in the context of CRF01_AE (53L) and subtype B (yielding a construct designated clone 19L). In our system, H87Q alone in NL4-3 did not confer resistance (Fig. 5), while the H87Q mutation in CRF01_AE significantly affects sensitivity of the virus to MxB restriction as previously shown. On the other hand, H87Q alone in NL4-3 conferred resistance to human TRIM5α in our system (Fig. 5), consistent with previously reported results.^34^

**FIG. 8.**
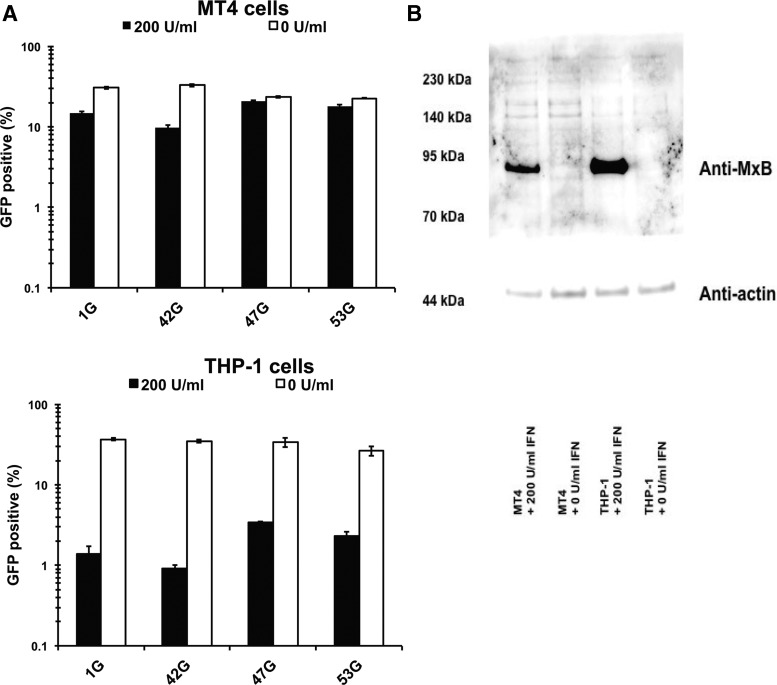
The effect of IFN-β treatment in myeloid cell line. MT4 and THP-1 cells were pretreated with 200 U/ml of IFN-β for 16 h and then infected with GFP-expressing HIV-1 with or without capsid mutations. We normalized input virus doses to obtain ∼30% GFP-positive cells at 2 days after infection. **(A)** *White* and *black bars* indicate percentage of GFP-positive cells (%) with and without IFN-β treatment, respectively. Data shown are mean of triplicate samples with SDs. The representative results of three independent experiments are shown. **(B)** Cells with or without 16 h IFN-β treatment were lysed for Western blotting analysis. Anti-MxB antibody and anti-actin antibody were mixed for protein detection at the same time. GFP, green fluorescence protein.

Finally, we examined the effect of type 1 IFN to the capsid mutant viruses, since it is known that MxB could be induced by type 1 IFN. As shown in Figure 6B, the expression levels of MxB were markedly increased after treatment with 200 U/ml in MT4 cells. We prepared GFP expressing viruses by transferring the *Bss*HII and Apa I fragment of pNL-Luci-R-E-, 42L, 47L (DTQ mutations on 42L), and 53L (H87Q mutation on 42L) to pMSMnG plasmid and designated 1G, 42G, 47G, and 53G, respectively. When GFP expressing viruses 1G and 42G were infected to MT4 or THP-1 cells treated with 200 U/ml of IFN-β, the virus infection was suppressed. Levels of virus suppression by IFN-β treatment were greater in THP-1 cells than in MT4 cells. As expected, the levels of IFN-β mediated virus suppression were almost completely diminished in MT4 cells and apparently reduced in THP-1 cells in the case of 47G (DTQ) and 53G (H87Q) viruses (Fig. 8A). These results suggested that the H87Q as well as DTQ mutations confer the type 1 IFN resistance in CRF01_AE. The expression levels of MxB protein in IFN-treated THP-1 cells were higher than that in MT4 cells, as shown in Figure 8B. This result is in a good agreement with our results (Fig. 8A) that the degree of viral suppression by IFN treatment was greater in THP-1 cells than in MT4 cells.

## Discussion

We found that the DTQ mutations in CRF01_AE capsid conferred partial resistance to MxB and human TRIM5α. Initially, our sequence analysis suggested that the DTQ mutations were present exclusively in the sequences of isolates from Beijing.^36^ However, when we performed a homology search (by BLAST) using the protein sequence DWDRTHPVQAGPIPPGQIREPRGSDIAGTTSNLQEQIAWMTN [corresponding to residues 79 to 120 of a capsid protein, including (as indicated by underlining) the DTQ mutations along with M96I, T110N, and G116A; clone 49L] as a query, multiple viruses with identical sequences were returned, as shown in Supplementary Table S3. All of these viruses sorted as CRF01_AE viruses and were isolated from cities in China, including Beijing, Guangxi, Jiangsu, and Shijiazhuang.

When we repeated the BLAST search using the protein sequence DWDRTHPVQ (corresponding to residues 79 to 87 of a capsid protein, including the DTQ mutations), identical virus sequences were returned from additional countries, including Thailand, Malaysia, Brazil, Cameroon, and Kenya (Supplementary Table S4). These results indicated that the combination of the DTQ mutations is already in worldwide circulation.

HIV transmission founder viruses are thought to have been relatively resistant to the effects of type 1 interferon (IFN). MxB and TRIM5α are known to be induced by the stimulation with type 1 IFN. One of the viruses with the DTQ mutations, KM217855, was reported as a transmission founder virus for the MSM outbreak in China, but the other founder viruses appear not to have harbored the DTQ mutations.^36^ Those results, in combination with the infection assays described in this study, indicate that resistance to MxB and human TRIM5α is not essential for transmission of HIV-1. Nevertheless, our results demonstrated that DTQ mutation conferred at least partial resistance against type 1 IFN (Fig. 8).

The introduction of the DTQ mutations in our reporter construct showed an apparent fitness cost in terms of virus infectivity. The detailed mechanism of this fitness cost is not clear at present. Since we did not obtain live virus isolated from patients, we do not know the replication competency of clinical isolates harboring DTQ mutations. Further analysis should include a comparison of the viral loads in patients infected with HIV-1 viruses with and without the DTQ mutations.

In our system, the H87Q substitution alone conferred substantial resistance to human MxB in the context of CRF01_AE, but not in the context of subtype B. The subtype-specific mechanism responsible for this difference is not clear at present. Our results suggest that the A92P mutation commonly found in CRF01_AE may play a supportive role when combined with H87Q. In conclusion, more attention should be paid to the role of the H87Q mutation in CRF01_AE infectivity.

As shown in Figure 8, the viral suppression levels induced by IFN were higher in THP-1 cells than in MT4 cells. Accordingly, levels of MxB expression in IFN-treated THP-1 cells were higher than in IFN-treated MT4 cells. Nevertheless, it would be interesting to investigate whether factors other than MxB are also involved in the effect of IFN in HIV-1 infected THP-1 cells,^37^ since both DTQ and H87Q mutations conferred nearly complete resistance against IFN in MT4 cells, while those mutant viruses were still potently suppressed by IFN (Fig. 8A).

Our analysis also suggested that there were multiple independent transmissions of HIV-1 CRF01_AE from Thailand to China. Future studies should examine HIV-1 sequences from surrounding countries such as Cambodia, Laos, Myanmar, and Vietnam; in combination with the sequences analyzed, such data are expected to permit elucidation of the precise routes whereby HIV-1 CRF01_AE spreads from Thailand.

## Supplementary Material

Supplemental data

Supplemental data

Supplemental data

Supplemental data

Supplemental data

Supplemental data

Supplemental data
