# Supplementary material for: Naturally Occurring Mutations in HIV-1 CRF01_AE Capsid Affect Viral Sensitivity to Restriction Factors
Source: AIDS Res Hum Retroviruses. 2018 Apr 1;34(4):382–92. doi: 10.1089/aid.2017.0212 (PMC5899301; doi:10.1089/aid.2017.0212)
Supplement: Supplemental data [file Supp_Table1.pdf]

Supplementary Data

Supplemental Table S1. List of References for Thai Sequences

Accession No.	*Title*	*Journal*
AB032740<br>AB032741	Full-length sequences of two CRF01_AE (subtype E) HIV type 1 isolates from 1995 samples of patients with sexually transmitted diseases in Thailand	AIDS Res Hum Retroviruses 17 (9), 867–871 (2001)
AY945712, AY945718<br>AY945726–AY945728<br>AY945731, AY945732<br>AY945740, AY945741	Molecular epidemiology of HIV type 1 in preparation for a phase III prime-boost vaccine trial in Thailand and a new approach to HIV type 1 genotyping	AIDS Res Hum Retroviruses 22 (8), 801–807 (2006)
AY358036–AY358052<br>AY358056, AY358057<br>AY358059, AY358060<br>AY358062–AY358073	The changing molecular epidemiology of HIV type 1 among northern Thai drug users, 1999 to 2002	AIDS Res Hum Retroviruses 20 (5), 465–475 (2004)
