# Supplementary material for: Naturally Occurring Mutations in HIV-1 CRF01_AE Capsid Affect Viral Sensitivity to Restriction Factors
Source: AIDS Res Hum Retroviruses. 2018 Apr 1;34(4):382–92. doi: 10.1089/aid.2017.0212 (PMC5899301; doi:10.1089/aid.2017.0212)
Supplement: Supplemental data [file Supp_Table2.pdf]

Supplemental Table S2. List of Reference for Chinese Sequences

| *Accession No.* | *Title* | *Journal* |
|---|---|---|
| JX112847, 49, 54, 55, 59<br>JX112796, 98, 99<br>JX112800-08 | The rapidly expanding CRF01_AE epidemic in China is driven by multiple lineages of HIV-1 viruses introduced in the 1990s | AIDS 27 (11), 1793–1802 (2013) |
| KM217833–KM217835<br>KM217837–KM217855 | Comprehensive characterization of the transmitted/founder *env* genes from a single MSM cohort in China | J Acquir Immune Defic Syndr 69 (4), 403–412 (2015) |
| JX960603–JX960626<br>JX960628–JX960634 | Reconstituting the epidemic history of CRF01_AE among MSM in Liaoning, northeastern China: implication in expanding MSM epidemic in China | J Virol 86 (22), 12402–12406 (2012) |
| KC183777 | New emerging recombinant HIV-1 strains and close transmission linkage of HIV-1 strains in the Chinese MSM population indicate a new epidemic risk | PLoS One 8 (1), E54322 (2013) |
| KJ778895<br>KJ778896 | Near full-length genomic characterization of a novel HIV type 1 CRF01_AE/07_BC recombinant in men who have sex with men from Shannxi, China | Unpublished |
