# Supplementary material for: Naturally Occurring Mutations in HIV-1 CRF01_AE Capsid Affect Viral Sensitivity to Restriction Factors
Source: AIDS Res Hum Retroviruses. 2018 Apr 1;34(4):382–92. doi: 10.1089/aid.2017.0212 (PMC5899301; doi:10.1089/aid.2017.0212)
Supplement: Supplemental data [file Supp_Table3.pdf]

Supplemental Table S3. BLAST Search Results with DWDRTHPVQAGPIPPGQIREPRGSDIAGTTSNLQEQIAWMTN

Accession No.	*Species*	*Transmission*	*Similarity*	*Location*	*Year*	*Title*	*Journal*
KF835542.1	CRF01_AE	Unknown	100%	Yunnan	2007	Characterization of near full-length genomes of HIV-1 in Yunnan, China: dominance of multiple circulating recombinant forms and unique recombinant forms	Unpublished
JQ900915.1, JQ900918.1, JQ900875.1	HIV-1	MSM	100%	Beijing	2008	Genetic characterization and transmitted drug resistance of the HIV type 1 epidemic in men who have sex with men in Beijing, China	AIDS Res Hum Retroviruses 29 (3), 633–637 (2013)
HQ215555.1, HQ215573.1	CRF01_AE	MSM	100%	Shijiazhuang	2009	High genetic diversity of HIV-1 was found in men who have sex with men in Shijiazhuang, China	Infect Genet Evol 11 (6), 1487–1492 (2011)
JX112796.1	CRF01_AE	Unknown	100%	Beijing	2010	The rapidly expanding CRF01_AE epidemic in China is driven by multiple lineages of HIV-1 viruses introduced in the 1990s	AIDS 27 (11), 1793–1802 (2013)
KF758551.1	CRF01_AE and B recombinant	MSM	100%	Jiangsu	2010	A novel HIV-1 CRF01_AE/B recombinant among men who have sex with men in Jiangsu Province, China	AIDS Res Hum Retroviruses 30 (7), 706–710 (2014)
JQ028668.1	CRF0	Unknown	100%	Guangxi	2010	Emergence of a new HIV type 1 CRF01_AE variant in Guangxi, Southern China	AIDS Res Hum Retroviruses 28 (10), 1352–1356 (2012)
JQ235004.1, JQ234985.1	HIV-1	MSM	100%	Zhengzhou	2010	Multiple introductions of HIV into men who have sex with men were found in Zhengzhou City, China	AIDS Res Hum Retroviruses 28 (9), 947–951 (2012)
KF818869.1, KF818864.1	CRF01_AE	Unknown	100%	Jilin	2011	Molecular epidemiology of HIV-1 in Jilin Province, Northeastern China: emergence of a new CRF07_BC transmission cluster and intersubtype recombinants	PLoS One 9 (10), e110738 (2014)
KC183782.1, KC183780.1	01B	MSM	100%	Anhui	2011	New emerging recombinant HIV-1 strains and close transmission<br>Linkage of HIV-1 strains in the Chinese MSM Population indicate a new epidemic risk	PLoS One 8 (1), e54322 (2013)
KR605017.1	CRF01_AE	MSM	100%	Hebei	2013	HIV-1 genetic diversity and transmitted drug resistance among recently infected individuals at men who have sex with men sentinel surveillance points in Hebei Province, China	AIDS Res Hum Retroviruses 31 (10), 1038–1045 (2015)
KR811276.1, KR811251.1	Unknown	Unknown	100%	Harbin	Unknown	Molecular epidemiology is becoming complex under the dynamic HIV prevalence: the perspective from Harbin, China	J Med Virol 88 (5), 807–814 (2016)

MSM, men who have sex with men.
