# Supplementary material for: Naturally Occurring Mutations in HIV-1 CRF01_AE Capsid Affect Viral Sensitivity to Restriction Factors
Source: AIDS Res Hum Retroviruses. 2018 Apr 1;34(4):382–92. doi: 10.1089/aid.2017.0212 (PMC5899301; doi:10.1089/aid.2017.0212)
Supplement: Supplemental data [file Supp_Table4.pdf]

Supplemental Table S4. BLAST Search Results with DWDRTHPVQ

Accession No.	*Type*	*Transmission*	*Similarity*	*Location*	*Year*	*Title*	*Journal*
ALF37197.1, ALF37187.1, ALF36900.1, ALF36996.1	CRF01_AE	Unknown	100%	China	Unknown	HIV-1 Thai B strains have spread out of former plasma donors into general population through sexual contact in Henan, China	Unpublished
AFV39337.1, AFV39352.1, AFV34206.1, AFV34197.1, AFV34188.1, AFV34170.1, AFV34162.1, AFV34153.1	HIV1	Unknown	100%	China	2009	Subtype CRF01_AE dominate the sexually transmitted human immunodeficiency virus type 1 epidemic in Guangxi, China	J Med Virol 85 (3), 388–395 (2013)
AJP61849.1	U	Unknown	100%	China	2013	Genomic characterization of two novel HIV-1 unique (CRF01_AE/B) recombinant forms among men who have sex with men in Beijing, China	AIDS Res Hum Retroviruses 31 (9), 921–925 (2015)
AJD23857.1	CRF01_AE	Unknown	100%	China	2009	CRF07_BC strain dominates the HIV-1 epidemic in injection drug users in Liangshan prefecture of Sichuan, China	AIDS Res Hum Retroviruses 31 (5), 479–87 (2015)
ACT76524.1, ACT76486.1, ACT76462.1, ACT76428.1	CRF01_AE	Unknown	100%	China	2008	Epidemiologic and genetic characterization of human immunodeficiency virus type I infection in Liaoning Province, China	Unpublished
AKI88290.1	HIV1	Unknown	100%	Kenya	1995	HIV-infected sex workers with beneficial HLA variants are potential hubs for selection of HIV-1 recombinants that may affect disease progression	Sci Rep 5, 11253 (2015)
ACA49250.1	HIV1	Unknown	100%	Brazil	2008 (issue)	High frequency of BF mosaic genomes among HIV-1-infected children from Sao Paulo, Brazil	Arch Virol 153 (10), 1799–1806 (2008)
AAG01668.1	HIV1	Unknown	100%	Nigeria	2000 (issue)	Spread of distinct human immunodeficiency virus type 1 AG recombinant lineages in Africa	J Gen Virol 81 (Pt. 2), 515–523 (2000)
AKP06682.1, AKP18465.1	CRF18_cpx	Unknown	100%	Cameroon	2011	Utility of unbiased next-generation sequencing for HIV surveillance	Unpublished
BAT68994.1	HIV1	Unknown	100%	Vietnam	2015	A strong association of HLA-associated Pol and Gag mutations with clinical parameters in HIV-1 subtype A/E infection	Unpublished
ACI45430.1	CRF01_AE	Unknown	100%	Thailand	2006	Impact of amino acid variations in Gag and protease of HIV type 1 CRF01_AE strains on drug susceptibility of virus to protease inhibitors	J Acquir Immune Defic Syndr 52 (3), 320–328 (2009)
ABV91559.1	HIV1	Unknown	100%	Malaysia	2007	Continuous crossover(s) events of HIV-1 CRF01_AE and B subtype strains in Malaysia: evidence of rapid and extensive HIV-1 evolution in the region	Curr HIV Res 6 (2), 108–116 (2008)
